# Effectiveness of off-the-shelf footwear in reducing foot pain in Australian Department of Veterans’ Affairs recipients not eligible for medical grade footwear: study protocol for a randomized controlled trial

**DOI:** 10.1186/1745-6215-14-106

**Published:** 2013-04-23

**Authors:** Hylton B Menz, Nicoletta Frescos, Shannon E Munteanu

**Affiliations:** 1 Lower Extremity and Gait Studies Program, Faculty of Health Sciences, La Trobe University, Bundoora, Victoria, 3086, Australia; 2 Department of Podiatry, Faculty of Health Sciences, La Trobe University, Bundoora, Victoria, 3086, Australia

## Abstract

**Background:**

Foot pain is highly prevalent in older people, and in many cases is associated with wearing inadequate footwear. In Australia, the Department of Veterans’ Affairs (DVA) covers the costs of medical grade footwear for veterans who have severe foot deformity. However, there is a high demand for footwear by veterans with foot pain who do not meet this eligibility criterion. Therefore, this article describes the design of a randomized controlled trial to evaluate the effectiveness of low cost, off-the-shelf footwear in reducing foot pain in DVA recipients who are currently not eligible for medical grade footwear.

**Methods:**

One hundred and twenty DVA clients with disabling foot pain residing in Melbourne, Australia, who are not eligible for medical grade footwear will be recruited from the DVA database, and will be randomly allocated to an intervention group or a ‘usual care’ control group. The intervention group will continue to receive their usual DVA-subsidized podiatry care in addition to being provided with low-cost, supportive footwear (Dr Comfort®, Vasyli Medical, Labrador, Queensland, Australia). The control group will also continue to receive DVA-subsidized podiatry care, but will not be provided with the footwear until the completion of the study. The primary outcome measure will be pain subscale on the Foot Health Status Questionnaire (FHSQ), measured at baseline and 4, 8, 12 and 16 weeks. Secondary outcome measures measured at baseline and 16 weeks will include the function subscale of the FHSQ, the Manchester Foot Pain and Disability Index, the number of DVA podiatry treatments required during the study period, general health-related quality of life (using the Short Form 12® Version 2.0), the number of falls experienced during the follow-up period, the Timed Up and Go test, the presence of hyperkeratotic lesions (corns and calluses), the number of participants using co-interventions to relieve foot pain, and participants’ perception of overall treatment effect. Data will be analyzed using the intention-to-treat principle.

**Discussion:**

This study is the first randomized controlled trial to evaluate the effectiveness of off-the-shelf footwear in reducing foot pain in DVA recipients. The intervention has been pragmatically designed to ensure that the study findings can be implemented into policy and clinical practice if found to be effective.

**Trial registration:**

Australian New Zealand Clinical Trials Registry: ACTRN12612000322831

## Background

Foot pain affects approximately one in three people over the age of 65 years [1-3] and has a significant impact on quality of life in this age-group. Studies of older people have demonstrated that foot pain is associated with decreased ability to undertake activities of daily living [4-9], problems with balance and gait [7,10,11] and an increased risk of falls [12-16]. Furthermore, several clinic-based studies assessing health-related quality of life across a range of age-groups have shown that people with foot disorders (such as generalized foot pain [2,17], nail infections [18-20], hallux valgus [21,22] hallux rigidus [23] and plantar heel pain [24]) demonstrate significantly lower scores than those without these conditions. A range of risk factors for foot problems has been identified, including increased age [25-27], female sex [7,8,26,28], obesity [8,10,28,29], and chronic conditions such as osteoarthritis and diabetes [8,10,29].

In addition to these risk factors, there is also strong evidence that many older people wear inappropriate or poor quality footwear, and that ill-fitting footwear may contribute to foot problems. A household survey of people aged over 80 years conducted in the United Kingdom found that most wore slippers all day, irrespective of whether they were housebound [30]. Similarly, a survey of the indoor shoe-wearing habits of 128 older people in Australia indicated that more than half spent less than 30 Australian dollars on their indoor footwear (most commonly slippers), replaced them infrequently, and often wore their indoor shoes for outdoor activities [31]. More recently, a survey of sub-acute aged care hospital patients reported that only 14% wore ‘safe’ footwear, with the most commonly observed detrimental features being a lack of fastening (86%), slippery soles (86%) and an excessively flexible heel counter (77%) [32].

By far the most commonly encountered problem with footwear in older people is the wearing of shoes that are too small. Burns *et al*. [33] compared the length and width of the feet and shoes of 65 people aged between 64 and 93 years attending a rehabilitation ward in the United Kingdom, and reported that 72% wore shoes of an incorrect size. Similarly, a study of 440 Veterans’ Affairs patients in the United States reported that only 26% were found to be wearing appropriately sized shoes [34], and a recent study of 213 people aged 60 to 80 years in Thailand reported that 50% of women and 34% of men wore shoes that were too narrow [35]. Several factors may be responsible for this, including fashion influences (particularly in older women [36,37]), not measuring foot dimensions when purchasing shoes [31], or the absence of commercially-available, low-cost footwear that adequately caters for the altered morphology of the elderly foot [38,39]. Irrespective of the underlying cause of poorly-fitting footwear, there is evidence that wearing shoes that are too small is associated with foot problems. In older people, wearing shoes substantially narrower than the foot is associated with corns on the toes, hallux valgus deformity and foot pain, whereas wearing shoes shorter than the foot is associated with lesser toe deformity [40]. Furthermore, a survey of 227 older women revealed that 61% reported foot pain when wearing shoes (most commonly in the forefoot and toes), and that those with foot pain exhibited a broader forefoot than those without pain [41].

In Australia, the Department of Veterans’ Affairs (DVA), as part of the Rehabilitation Appliances Program, covers the costs of medical grade footwear for veterans who have severe foot deformity (defined as feet that cannot be accommodated in regular ‘off-the-shelf’ footwear). Footwear provision is one of the most common podiatry interventions. An analysis of 1996 to 1997 DVA data indicated that out of the total podiatry DVA population who had received a podiatry intervention (n = 4,418), 3,227 (73%) received new medical grade footwear [42]. However, there is also a high demand for footwear by veterans who have foot pain but do not have severely deformed feet. In this context, it is likely that there are a substantial number of veterans who could benefit from the provision of more appropriate footwear. Such an intervention may also reduce the need for frequent, on-going maintenance foot care provided by podiatrists under the DVA scheme.

Therefore, the aim of this study is to evaluate the effectiveness of relatively low-cost but good quality, custom-fitted footwear to DVA clients with foot pain who do not currently meet the structural deformity criteria for medical grade footwear.

## Methods

The trial has been registered on the Australian New Zealand Clinical Trials Registry (ACTRN12612000322831).

### Ethical approval

The Australian DVA Human Research Ethics Committee provided ethical approval (approval number E012/005[5.1]) and the La Trobe University Human Ethics Committee formally accepted this approval (E012/004). All participants will provide written informed consent prior to enrolment. Ethical standards will adhere to the National Health and Medical Research Council (NHMRC) National Statement [43] and the World Medical Association's Declaration of Helsinki [44]. Publications associated with the trial will be formatted according to the Consolidated Standards of Reporting Trials (CONSORT) 2010 statement [45,46].

## Design

**Figure 1:**
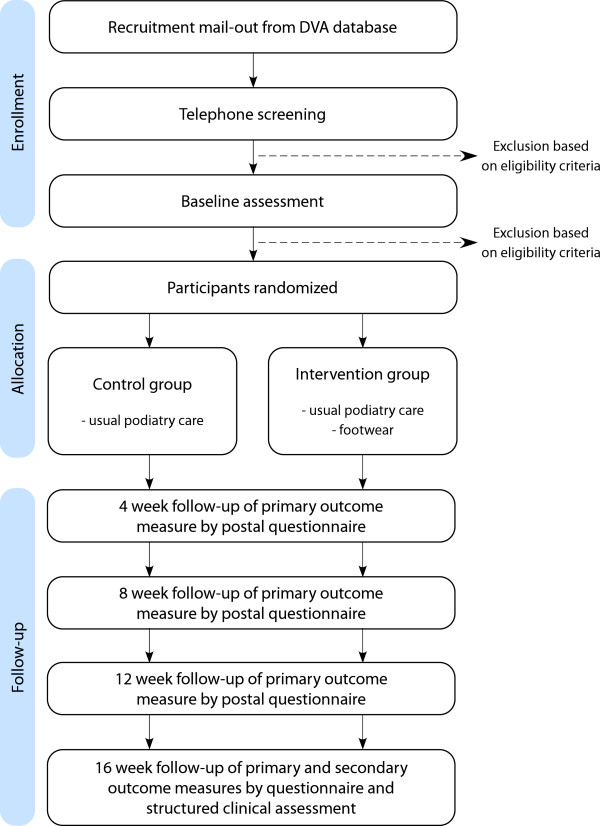
Trial profile.

This study is a two-group randomized controlled trial with a 16 week prospective follow-up (Figure 1). Participants will be randomly allocated to either a ‘usual care’ control group or the intervention group [47]. Permuted block randomization with random block sizes (stratified by sex [48]) will be undertaken using an interactive voice response telephone service provided by the NHMRC Clinical Trials Centre at the University of Sydney, New South Wales, Australia to ensure allocation concealment. Due to the nature of the intervention, it will not be possible to blind the participants or investigators. However, the primary outcome measure is administered as a self-completion questionnaire and will be entered into the database by an investigator blinded to group allocation.

### Participant recruitment, screening and eligibility criteria

The geocoding feature of the DVA database will be used to identify veterans currently receiving podiatry treatments who are residing in Melbourne, Victoria. From this group of veterans, the DVA Departmental Management Information System will be used to identify those who have not been issued with medical grade footwear within the last five years, by excluding veterans who have documented a footwear-related item number during this period (item numbers F604, F605, F606, F611, F612, F625, F660, F661 F670, F671, F615 or F616). The remaining veterans will form the primary recruitment source and will be mailed an information package about the study, along with an invitation to contact the research team by either return mail or by telephone. Database recruitment will be complemented by running advertisements in DVA newsletters and placing posters at Returned and Services Leagues Clubs in the surrounding suburbs.

During the initial telephone contact with participants, a member of the research team will determine each veteran’s eligibility by structured interview. To be included in the study, veterans will need to meet the following inclusion criteria:

(i) be aged 65 years or over;

(ii) be a current DVA Gold Card client not eligible for   medical grade footwear;

(iii) have received podiatry treatment on at least three   occasions in the past five years;

(iv) have disabling foot pain, using the case definition   of  the Manchester Foot Pain and Disability Index   (MFPDI) [49] proposed by Roddy *et al*. [50]. The   MFPDI consists of 19 statements prefaced by the   phrase ‘Because of pain in my feet’, formalized   under three constructs: functional limitation (10   items), pain intensity (five items), and personal   appearance (two items), with three possible   answers: ‘none of the time’ (score = 0), ‘some days’   (score = 1), and ‘most days/every day’ (score = 2).   The Roddy *et al*. definition requires at least one of   the ten functional limitation items to be documented   on most/every day(s) in the last month;

(v) have persistent foot pain, defined as foot pain   present for at least 12 weeks, and;

(vi) be capable of understanding the English language   in verbal and written form.

Veterans will not be eligible for inclusion in the study if they:

(i) are currently residing in a residential aged care   facility;

(ii) have diabetes and a history of foot ulceration (or   current foot ulceration) or diabetic peripheral   neuropathy (diagnosed with the 5.07 Semmes- Weinstein monofilament, using the International   Working Group on the Diabetic Foot protocol [51]);

(iii) have a neurodegenerative disorder (for example,   Parkinson’s disease);

(iv) have had a lower limb or partial foot amputation   (although single toe amputations will be permitted);

(v)  have been prescribed contoured foot orthoses   within the past three months (although simple flat   insoles will be permitted, as will contoured foot   orthoses prescribed more than three months ago);

(vi) are currently wearing the intervention footwear, or;

(vii)  have cognitive impairment (defined as a score of <   7 on the Short Portable Mental Status   Questionnaire [52]).

### Intervention group

**Figure 2:**
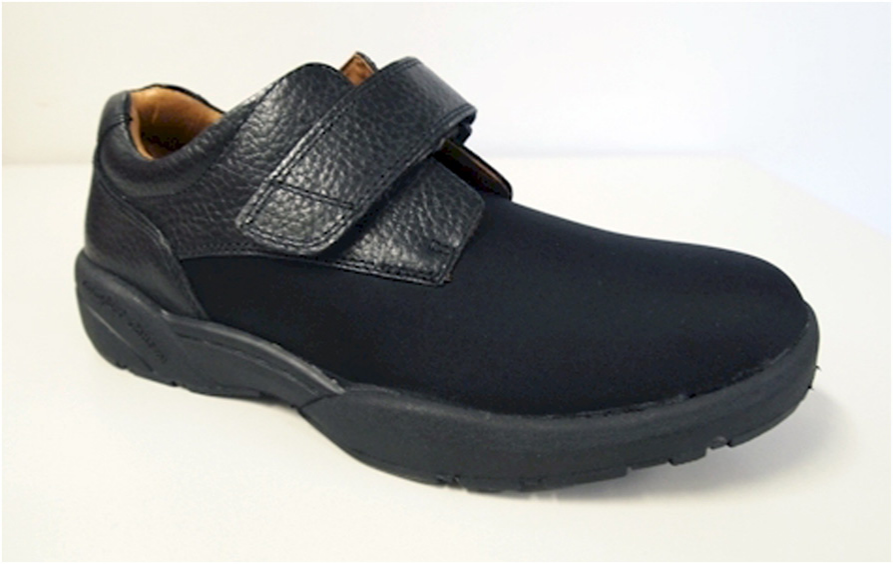
Dr Comfort® footwear to be used in the study for men (‘Brian’ style).

**Figure 3:**
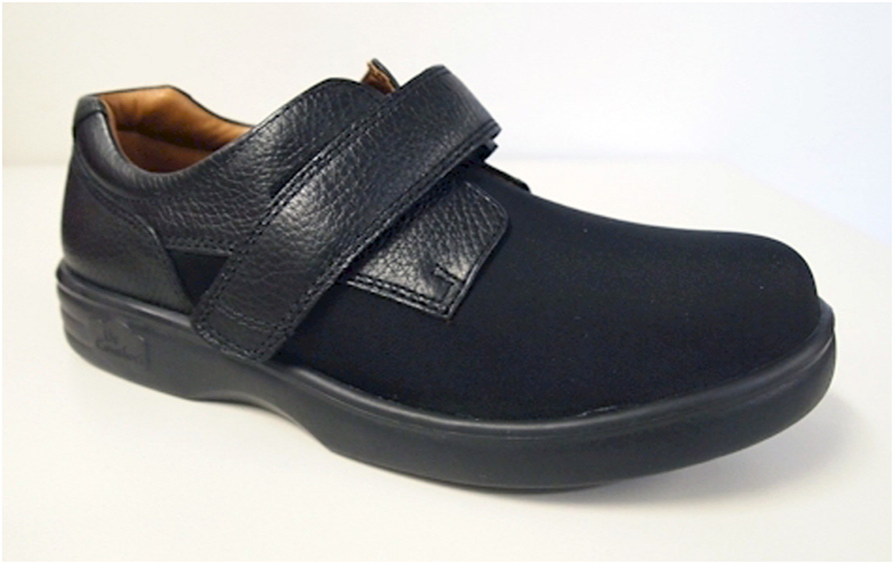
Dr Comfort® footwear to be used in the study for women (‘Annie’ style).

The intervention group will continue to receive regular podiatry treatment as clinically required. This will typically include toenail maintenance and scalpel debridement of hyperkeratotic lesions (corns and calluses). In addition, they will be provided with good quality, off-the-shelf footwear (Dr Comfort® Vasyli Medical, Labrador, Queensland, Australia). Men will receive the ‘Brian’ style and women will receive the ‘Annie’ style (see Figures 2 and 3). Both styles are available in three width fittings (medium, wide, extra-wide) and feature a stretchable Lycra® upper with Velcro® closure and a removable, flat cushioned insole. These shoes have been selected as they meet all commonly-used criteria for appropriate footwear (such as a low heel, appropriate fixation and adequate depth to accommodate toe deformities) [53-57]. Due to sex differences in foot dimensions (and therefore the dimensions of the lasts the shoes are constructed from) the ‘Brian’ style has a relatively broader fit than the ‘Annie’ style. However, both the heel height and toe spring are the same for shoes of equivalent length across the two styles.

The research staff will measure participants’ feet using a Brannock Device® (Brannock Device Co, Inc., Liverpool, New York, USA) to ensure appropriate length and width, using the fitting protocol recommended by the footwear manufacturer [58]. Intervention group participants who wear flat insoles (or have been wearing contoured foot orthoses for more than three months) in their current footwear will be permitted to wear them in their study footwear, provided that the fit of the shoes is considered to be appropriate.

### Control group

The control group will continue to receive regular podiatry treatment as clinically required. This will typically include toenail maintenance and scalpel debridement of hyperkeratotic lesions (corns and calluses). Upon completion of the trial, they will be provided with the same footwear used in the intervention group.

### Baseline assessments

Participant characteristics will be collected by structured interview at the baseline assessment and will include age, sex, height, weight, waist circumference, hip circumference, country of birth, education, major medical conditions, medications, use of walking aids and cigarette smoking history. The following questionnaires and clinical tests will also be administered:

(i) foot pain characteristics, including duration (in months) and location, using a standardized foot diagram;

(ii) presence and severity of hallux valgus, assessed using the Manchester Scale [59];

(iii) foot structure and presence of hyperkeratotic lesions (corns and calluses), documented with three-dimensional clinical photographs using the FotoScan™ 3D foot scanner (Precision 3D Limited, Weston-super-Mare, UK);

(iv) the Pain Catastrophizing Scale, a questionnaire containing 13 items reflecting elevated negative cognitive responses to pain [60];

(v) the short version of the Geriatric Depression Scale, a 15-item depression screening tool that has been specifically validated in older people [61,62];

(vi)  footwear assessment, using selected components of the recently developed Footwear Assessment Tool [57], and;

(vii)  physical activity levels, using the Incidental and Planned Activity Questionnaire [63].

In addition, participants allocated to the footwear intervention group will be asked to complete six 100 mm visual analogue scales to ascertain their perceptions of: (i) the immediate level of comfort of the footwear (using the anchors ‘extremely uncomfortable’ and ‘extremely comfortable’ [64]); (ii) the attractiveness of the footwear (using the anchors ‘extremely unattractive’ and ‘extremely attractive’); (iii) how attractive they think other people would find the footwear (using the anchors ‘extremely unattractive’ and ‘extremely attractive’); (iv) how well the shoes fit (using the anchors ‘poorest fit possible’ and ‘best fit possible’); (v) how easy it is to put the shoes on and take them off (using the anchors ‘most difficult as possible’ and ‘as easy as imaginable’) and; (vi) how heavy the shoes are (using the anchors ‘extremely light’ and ‘extremely heavy’).

The perceived therapeutic value of the shoes will be assessed using responses to the statement ‘I believe that the shoes provided to me can reduce the severity of my foot pain’, with a five-point Likert scale ranging from ‘strongly disagree’ to ‘strongly agree’. In addition, two 100 mm visual analogue scales will be used to ascertain expectations of pain reduction associated with the footwear, with the questions ‘With your shoes, do you expect to have less or more pain in the skin of your feet and/or ankles, during activities like standing and/or walking?’ and ‘With your shoes, do you expect to have less or more pain in the muscles and joints of your feet and/or ankles, during activities like standing and/or walking?’, both using the anchors ‘much less’ and ‘much more’. These questions are derived from the Monitor Orthopedic Shoes questionnaire [65], and will be used to predict adherence to, and effectiveness of, the footwear intervention at the completion of the study.

### Primary outcome measure

The primary outcome measure will be the pain domain of the Foot Health Status Questionnaire (FHSQ). The FHSQ consists of 13 questions reflecting four foot health-related domains: foot pain, foot function, footwear, and general foot health [66]. The FHSQ pain domain comprises four questions, with higher scores representing better foot health (that is, 100 = best foot health and 0 = worst foot health). The FHSQ pain domain demonstrates a high degree of content, criterion, and construct validity (Cronbach α = 0.88), high retest reliability (intraclass correlation coefficient = 0.86) and has been used as an outcome measure in clinical trials for a range of foot disorders [67]. Previous research indicates that the minimal important difference for this measure is 12.5 points [68]. The FHSQ pain domain will be measured at baseline and at 4, 8, 12 and 16 weeks, with the primary endpoint being the 16 week score.

### Secondary outcome measures

Secondary outcome measures will be documented at baseline and 16 weeks and will include:

(i) the function domain of the FHSQ [3];

(ii) the functional limitation, pain intensity and concern about appearance subscales of the MFPDI [49];

(iii) the number of DVA podiatry treatments required during the study period;

(iv) general health-related quality of life, assessed with the Short Form 12® Version 2.0 [69];

(v) the number of falls experienced during the follow-up period;

(vi) functional mobility, using the Timed Up and Go test [70];

(vii) presence of hyperkeratotic lesions (corns and calluses);

(viii) number of participants using co-interventions to relieve foot pain (such as oral non-steroidal antiinflammatory medications, topical medications and visits to other health-care practitioners), documented using a diary at 4, 8, 12 and 16 weeks, and;

(ix) participants’ perception of overall treatment effect at week 16, assessed with the question ‘Overall, how has your foot pain changed since the start of the study?’ with a five-point Likert scale response (‘marked worsening’, ‘moderate worsening’, ‘same’, ‘moderate improvement’, or ‘marked improvement’). For the purpose of analysis, this scale will then be dichotomized, where ‘success’ is defined as marked or moderate improvement on this scale.

### Sample size

The sample size for the study has been calculated based on the pain domain of the FHSQ as the primary outcome measure [66]. Using a minimal important difference of 12.5 and a standard deviation of 23 obtained from a previous study [68], and assuming a 10% drop-out rate, the required sample size is 60 per group (power = 80%). We believe that the 10% drop-out rate is reasonable, as our recent randomized controlled trial of a multifaceted podiatry intervention to prevent falls in older people had a drop-out rate of less than 5% over a much longer period of follow-up (12 months) [71]. The extra precision provided by covariate analysis was conservatively ignored when performing this calculation.

### Evaluation of adherence

Adherence to the intervention will be documented at 4, 8, 12 and 16 weeks by asking participants on how many days (and for how many hours) they have worn their footwear, on average, in the past month.

### Complications and adverse events

Complications and adverse events associated with the intervention are unlikely. However, the questionnaires at the 4, 8, 12 and 16 week follow-ups will provide participants with an opportunity to report any difficulties they have with the footwear, and all adverse events will be reported in the final manuscript.

### Statistical analysis

Statistical analysis will be undertaken using SPSS version 20.0 (IBM Corp, NY, USA). All analyses will be conducted on an intention-to-treat principle using all randomized participants. Multiple imputation will be used to replace any missing data using five iterations, with age, baseline scores, and group allocation as predictors [72]. Demographic characteristics and baseline data will be summarized by descriptive statistics. Continuous data will be explored for normality using standard tests to satisfy the assumptions of parametric statistics. If data are found to be not normally distributed, transformation will be attempted using conventional techniques [73]. However, if data are still not normally distributed after transformation, non-parametric statistical tests will be used. Continuously-scored primary and secondary outcome measures with a normal distribution will be compared between groups using a linear regression technique with baseline scores, adjusted for by the analysis of covariance model [74,75]. Nominal and ordinal scaled data will be compared using chi-square analyses (or Fisher's exact test where appropriate) and Mann–Whitney *U*-tests, respectively. Effect sizes will be determined using Cohen’s *d*[76] for continuous data and relative risks for nominal data. Statistical significance for hypothesis tests will be set at the conventional level of α = 0.05.

## Discussion

Footwear plays an important role in the maintenance of foot health and mobility in older people, and it has long been recognized that suboptimal footwear can be detrimental [77,78]. The provision of appropriate footwear, therefore, has considerable potential to reduce pain and improve health-related quality of life in this age-group. Although several studies have been undertaken to assess the effectiveness of footwear interventions in reducing foot pain in people with rheumatoid arthritis and other degenerative foot disorders [79-81], none have focused on the general veteran community. The study outlined in this protocol is, therefore, novel in that it targets a specific group of veterans (those ineligible for medical grade footwear) and uses generic, off-the-shelf footwear as the intervention.

Participants will be followed up at four-weekly intervals up to 16 weeks. This is longer than previous footwear intervention studies, which have employed 8 week [79] or 12 week [80,81] follow-up periods. We are, therefore, confident that the duration of the study is sufficient to detect differences between the groups, if they exist. Furthermore, previous footwear studies have been limited to single baseline and follow-up assessments [79-81], whereas our study will incorporate five repeated measurements for the primary outcome measure (baseline, 4 weeks, 8 weeks, 12 weeks and 16 weeks). This will provide additional insight into the trajectory of any improvements in participants’ foot pain. However, to address the issue of multiple testing of serial measurements [82,83], we have pre-specified 16 weeks as the primary end-point, and no statistical comparisons of the 4, 8 and 12 week scores will be undertaken.

Poor adherence is a well-recognized problem with footwear intervention studies [71,80,84] and has been attributed to the unique role of footwear as both an item of clothing and a health-related intervention [85]. We expect that the level of adherence to the off-the-shelf footwear in this study will be higher than that associated with medical grade footwear, due to better cosmesis. Furthermore, the initial postal invitation to take part in the study will specify the type of footwear that will be provided (accompanied by photographs), thereby providing veterans with an opportunity to decline participation on the basis of dissatisfaction with the appearance of the footwear. However, it is still likely that some participants will not be satisfied with the footwear either at the point of issue or during the follow-up period. The inclusion of baseline questionnaires addressing participants’ immediate perceptions of the attractiveness, comfort and usability of the footwear may provide useful insights into their adherence during the trial.

There are three key limitations of the study design that warrant careful consideration. Firstly, due to the nature of the intervention, it will not be possible to blind participants to their group allocation, nor will it be possible to blind the investigators providing the intervention. Secondly, because the ‘usual care’ control group will receive no additional treatment until the completion of the study, there is a risk of what is often referred to as ‘resentful demoralization’. That is, participants allocated to the control group may be resentful of not receiving the intervention, which may affect their adherence to the tasks required of them in the study and systematically influence their responses to the outcome measure questionnaires [86]. While this cannot be completely avoided, it may be offset to some extent by the fact that the control group participants will receive the intervention footwear at the completion of the trial, free of charge - footwear that they would not otherwise be eligible for under the DVA program. One suggested approach to address this issue is to document participant group allocation preference as a prognostic variable and consider this in the statistical analysis [87]. However, this would not be appropriate in our study, as it is unlikely that any participants would prefer to wait 16 weeks for their footwear rather than receiving it at the commencement of the study. Finally, this trial has been pragmatically designed to ensure that the findings can be easily translated into ‘real world’ situations, where off-the-shelf footwear is most often purchased without clinical assessment or diagnosis from foot health professionals. As such, we have not restricted the inclusion criteria in relation to the underlying *cause* of foot pain, so it is possible that our sample will include some participants with conditions that may not be amenable to treatment with footwear.

## Trial status

In summary, this study will be the first randomized controlled trial investigating the effectiveness of off-the-shelf footwear in reducing foot pain in Australian DVA clients ineligible for medical grade footwear. The study findings will be used to make evidence-based recommendations regarding the potential role of this intervention and will help inform the footwear provision component of the Rehabilitation Appliances Program of the DVA. Recruitment of participants commenced in October 2012 and final results are expected to be available in November 2013.

## Abbreviations

DVA: Department of Veterans’ Affairs; FHSQ: Foot Health Status Questionnaire; MFPDI: Manchester Foot Pain and Disability Index.

## Competing interests

The authors declare that they have no competing interests.

## Authors’ contributions

HBM and NF conceived the idea and obtained funding for the study. HBM, NF and SEM designed the trial protocol. HBM drafted the manuscript. All authors have read and approved the final manuscript.
